# Preliminary Data on *Silybum marianum* Metabolites: Comprehensive Characterization, Antioxidant, Antidiabetic, Antimicrobial Activities, LC-MS/MS Profiling, and Predicted ADMET Analysis

**DOI:** 10.3390/metabo15010013

**Published:** 2025-01-03

**Authors:** Sabrina Lekmine, Ouided Benslama, Mohammad Shamsul Ola, Nabil Touzout, Hamza Moussa, Hichem Tahraoui, Haroun Hafsa, Jie Zhang, Abdeltif Amrane

**Affiliations:** 1 Biotechnology, Water, Environment and Health Laboratory, Abbes Laghrour University, Khenchela 40000, Algeria; 2 Laboratory of Natural Substances, Biomolecules, and Biotechnological Applications, Department of Natural and Life Sciences, Larbi Ben M’Hidi University, Oum El Bouaghi 04000, Algeria; 3 Department of Biochemistry, College of Science, King Saud University, Riyadh 11451, Saudi Arabia; 4 Laboratory of Biomaterials and Transport Phenomena (LBMTP), University Yahia Fares, Médéa 26000, Algeriahichemm.tahraouii@gmail.com (H.T.); 5 Laboratoire de Gestion et Valorisation des Ressources Naturelles et Assurance Qualité (LGVRNAQ), Faculté des Sciences de la Nature et de la Vie et des Sciences de la Terre, Université de Bouira, Bouira 10000, Algeria; 6 Département des Sciences Biologiques, Faculté des Sciences de la Nature et de la Vie et des Sciences de la Terre, Université de Bouira, Bouira 10000, Algeria; 7 Laboratoire de Génie des Procédés Chimiques, Département de Génie des Procédés, Faculté de Technologie, Université Ferhat Abbas, Sétif-1, Sétif 19000, Algeria; 8 Univ Rennes, Ecole Nationale Supérieure de Chimie de Rennes, CNRS, ISCR—UMR6226, 35000 Rennes, France; 9 Laboratory of Reaction Engineering, USTHB, BP 32, Algiers 16111, Algeria; 10 School of Engineering, Merz Court, Newcastle University, Newcastle upon Tyne NE1 7RU, UK

**Keywords:** *Silybum marianum*, antioxidant, enzyme-inhibitory, antimicrobial, microwave-enhanced extraction

## Abstract

Background/Objectives: *Silybum marianum* extract, obtained via microwave-enhanced extraction, was evaluated for its antioxidant, antidiabetic, and antimicrobial activities to explore its therapeutic potential. Methods: The extraction was performed using microwave-enhanced techniques, and LC-MS/MS was employed to profile the metabolites in the extract. Total phenolic and flavonoid contents were quantified using spectrophotometric methods. Antioxidant activity was assessed using DPPH, ABTS, CUPRAC, Phenanthroline, and FRAP assays. Enzyme inhibition assays were conducted to evaluate antidiabetic activity against α-glucosidase and α-amylase. Antimicrobial activity was determined using the disc diffusion method, and in silico ADMET and drug-likeness analyses were performed for key metabolites. Results: The extract contained 251.2 ± 1.2 mg GAE/g of total phenolics and 125.1 ± 1.6 mg QE/g of total flavonoids, with 33 metabolites identified, including phenolic acids, tannins, flavonoids, and flavolignans. Strong antioxidant activity was observed, with IC_50_ values of 19.2 ± 2.3 μg/mL (DPPH), 7.2 ± 1.7 μg/mL (ABTS), 22.2 ± 1.2 μg/mL (CUPRAC), 35.2 ± 1.8 μg/mL (Phenanthroline), and 24.1 ± 1.2 μg/mL (FRAP). Antidiabetic effects were significant, with IC_50_ values of 18.1 ± 1.7 μg/mL (α-glucosidase) and 26.5 ± 1.3 μg/mL (α-amylase). Antimicrobial activity demonstrated inhibition zones of 8.9 ± 1.1 mm (*Bacillus subtilis*), 12.6 ± 1.6 mm (*Escherichia coli*), 8.2 ± 1.2 mm (*Fusarium oxysporum*), and 9.2 ± 1.1 mm (*Aspergillus niger*). In silico analyses showed high absorption, favorable metabolism and excretion, and minimal toxicity, with no hERG channel inhibition or hepatotoxicity. Conclusions: The comprehensive results highlight the significant antioxidant, antidiabetic, and antimicrobial activities of *S. marianum* extract, suggesting its potential for therapeutic and preventive applications.

## 1. Introduction

Diabetes mellitus, a chronic metabolic disorder, is primarily marked by prolonged elevated blood glucose levels (chronic hyperglycemia) due to the body’s failure to produce or efficiently utilize insulin, a crucial hormone for maintaining blood sugar balance [1]. If untreated, diabetes can lead to severe complications including vision impairment (blindness), chronic kidney disease (kidney failure), neuropathy (nerve damage), and cardiovascular ailments. It is categorized into type 1 and type 2 diabetes [2]. Type 1 diabetes is an autoimmune condition where the organism targets and destroys pancreatic beta cells that produce insulin [3]. Type 2 diabetes, which constitutes 90–95% of cases, is characterized by insulin resistance. Diagnosing diabetes typically involves laboratory measurements of glucose concentration, such as oral glucose tolerance test, fasting blood glucose, or glycated hemoglobin (HbA1c) values [4]. Management requires a combination of lifestyle adjustments, including diet changes, consistent physical activity, and maintaining a healthy weight, along with medical treatments like insulin therapy or oral antidiabetic drugs, tailored to the type and severity of the disease [5]. Ongoing research aims to develop more effective treatments, improve management strategies, and ultimately find a cure for this debilitating condition. Researchers are increasingly focusing on herbal remedies due to their effectiveness, availability, and typically fewer side effects compared to synthetic drugs [6]. Across both developed and underdeveloped countries, these herbs have been utilized since ancient times as traditional remedies for a variety of physiopathological conditions, such as diabetes mellitus. Therapeutic plants offer a good source of bioactive molecules that can provide healing properties [4]. To fully harness the healing properties of herbal remedies, it is essential to utilize eco-friendly extraction methods like microwave-enhanced extraction (MEE) [7]. MEE plays a crucial role in obtaining a higher yield of phenolic compounds from plant materials due to its efficiency and effectiveness. This technique utilizes microwave energy to heat both the solvent and the plant material rapidly and uniformly, disrupting plant cell walls and enhancing the extraction of phenolic compounds into the solvent. MEE significantly reduces the time of extraction compared to traditional methods and often results in higher yields of phenolic compounds [8]. Additionally, MEE can improve the quality of the extract by minimizing the degradation of heat-sensitive phenolic compounds. The rapid heating and controlled extraction conditions provided by MEE make it an optimal technique for extracting phenolic compounds from plants, resulting in extracts rich in phenolic compounds with enhanced antioxidant and bioactive properties [9]. Furthermore, the use of eco-friendly methods like MEE ensures sustainability and reduces environmental impact, aligning with the principles of green chemistry by reducing solvent usage and energy consumption [10]. This integration of traditional medicinal knowledge with modern, sustainable extraction technologies represents a promising avenue for developing effective and environmentally responsible therapeutic agents [10]. The vast array of the plant kingdom encompasses numerous species with therapeutic properties, among which is *Silybum marianum* (Family: Asteraceae, Genus: *Silybum*) [11]. This plant is abundant in bioactive compounds such as phenolic acids, flavonoids, tannins, terpenoids, and saponins [12]. These constituents contribute to a range of biological activities, including antioxidant, antifungal, and antimicrobial effects, as demonstrated in previous research [13,14]. Moreover, *S. marianum* is recognized for its hepatoprotective, anti-inflammatory, and immunomodulatory effects, as well as its potential in diabetes management [15]. The antidiabetic, antioxidant, and antimicrobial activities of plant extracts are interconnected, with each activity supporting and enhancing the others. Antioxidants can protect pancreatic beta cells from oxidative stress, contributing to antidiabetic effects, while also scavenging reactive oxygen species (ROS) generated by microorganisms, thereby enhancing antimicrobial activity. This multifaceted approach can provide a comprehensive therapeutic strategy for treating various diseases and conditions. This research aims to explore the antioxidant, antidiabetic, and antimicrobial processes of *S. marianum* extract obtained by microwave-enhanced extraction (MEE), and to evaluate the plant’s inhibitory effects on the α-glucosidase and α-amylase enzymes, which are crucial in diabetes management.

Several studies in the literature have explored the bioactive properties of *S. marianum*. However, our study distinguishes itself through several innovative aspects. Firstly, we utilized a microwave-enhanced extraction method, unlike the conventional extraction methods commonly used. Additionally, our LC-MS/MS analysis provides a much more exhaustive profile compared to previous studies. This analysis was conducted to confirm whether the extraction method we used for this plant was indeed effective, and it showed that this method yielded a significantly high output, thus validating its efficacy.

Regarding antioxidant activity, our study evaluated new methods, including the DPPH and ABTS assays, demonstrating high efficacy. Unlike previous studies that often used more traditional methods and reported higher IC_50_ values, our approach provides a more detailed and precise view of the antioxidant efficacy of the extracts. We also conducted comprehensive tests of reducing power, including CUPRAC, Phenanthroline, and FRAP tests, while previous studies often employed less detailed or varied tests.

Another innovative aspect of our study is the inclusion of in silico tests, particularly ADMET analysis (Absorption, Distribution, Metabolism, Excretion, and Toxicity). These analyses play a crucial role in evaluating the pharmacokinetic and toxicological properties of the compounds, allowing us to predict their behavior in the human body. None of the previous studies include this aspect, which enhances the relevance and originality of our work.

These differences highlight the unique contribution of our study in the research field on *S. marianum* and reinforce its therapeutic potential.

## 2. Materials and Methods

### 2.1. Plant Material and Microwave-Enhanced Extraction

*S. marianum* was freshly harvested. The identification of the plant was confirmed by Professor Zraib, a botanist from Khenchla University.

The plants were collected from Oum El Bouaghi, Algeria. This region is characterized by a Mediterranean climate with dry summers and mild, wet winters. The habitat consists of open fields and rocky slopes with sandy-loam soil, which supports a diverse range of native flora.

The plant parts were then meticulously washed to remove any surface contaminants. After cleaning, the materials were allowed to air dry at ambient temperature in a well-ventilated space, away from direct sunlight. The desiccation of the plant components required a period ranging from two to six weeks, following which residual contaminants were eliminated, and the material was pulverized to achieve homogeneity for subsequent analyses.

Ten TFM Teflon closed containers were used for microwave-enhanced extraction utilizing an ETHOS 1 microwave oven (Milestone, Shelton, CT, USA), which was managed by an ATC-400FO auto fiber optic temperature controller. The extraction involved 200 W microwave power and 50 °C temperature, using a magnetic stir bar, ethanol/water mixture, and filtering. The extraction was conducted in triplicate to ensure consistency.

### 2.2. Total Bioactive Compounds

The entire phenolic content of the *S. marianum* extract was assessed using the Folin–Ciocalteu spectrophotometric technique (Sigma-Aldrich, Steinheim, Germany) involving the mixing of Folin–Ciocalteu reagent and sodium carbonate solution, with absorbance measured at 765 nm. The total flavonoid content was determined using the aluminum chloride spectrophotometric assay, which included combining the extract with potassium acetate and aluminum chloride, and measuring the absorbance at 415 nm [16,17].

### 2.3. Method of LC-MS/MS for Identifying Phenolic Molecules

The identification of phenolic compounds, flavonoids, and tannins in *S. marianum* were carried out using advanced analytical techniques (LC-MS/MS from Corporation, Kyoto, Japan). The plant samples were first subjected to microwave-enhanced extraction (MEE) with an ethanol–water mixture. The extracts were subsequently passed through a 0.22 µm membrane filter, concentrated under reduced pressure. The extracts were subsequently dissolved in methanol for LC-MS/MS evaluation. A C18 reverse-phase column was used during the LC-MS/MS investigation. Solvents A (water with 0.1% formic acid) and B (acetonitrile with 0.1% formic acid) were employed in the gradient chromatography system. The column was maintained at an ambient temperature, and the injection volume was controlled. Mass spectrometry was conducted on a triple quadrupole instrument using negative ion electrospray ionization (ESI-) [18].

### 2.4. Antioxidant Activities

The antioxidant potential was evaluated using various methods, including Cupric Reducing Antioxidant Capacity (CUPRAC), O-Phenanthroline Analytical Method, ABTS, DPPH, and FRAP [19].

#### 2.4.1. Cupric Reducing Antioxidant Capacity (CUPRAC) Assay

The reduction of copper ions was determined using the CUPRAC method described by Apak et al. (2004). In brief, test solutions were prepared by mixing 50 μL of 10 mM Cu(II), 50 μL of 7.5 mM neocuproine, and 60 μL of 1 M NH4Ac buffer solution (pH = 7.0). Different concentrations of extracts were added to this initial mixture to achieve a final volume of 200 μL in each well of the microplate. After 1 h, absorbance was measured at 450 nm. The results were calculated as A0.5 (μg/mL), corresponding to the concentration indicating 0.50 absorbance, and the reducing capacity was compared to those of α-tocopherol and BHT [20].

#### 2.4.2. DPPH Free Radical Scavenging Assay

The 1,1-Diphenyl-2-picrylhydrazyl (DPPH) radical scavenging activity of the n-butanol fraction was assessed using a modified version of a previously established method [21]. The concentration causing 50% inhibition (IC_50_) was determined and compared with antioxidant standards such as BHA, BHT, Trolox, and ascorbic acid.

#### 2.4.3. ABTS Cation Radical Assay

The free radical scavenging activity of ABTS was evaluated using a slightly modified version of the method described in [22]. Butylated hydroxytoluene (BHT) served as the positive control. Results are expressed as the 50% inhibitory concentration (IC_50_).

#### 2.4.4. Ferric Reducing Antioxidant Power (FRAP) Assay

The FRAP assay is widely employed to determine the antioxidant capacity of various substances, including foods, beverages, and biological samples. This technique involves the reduction of a ferric tripyridyltriazine (Fe^3+^-TPTZ) complex to its ferrous form (Fe^2+^), resulting in a distinctive blue color. The intensity of this color is measured spectrophotometrically, with the antioxidant capacity reported as Trolox equivalents, a vitamin E analog [22].

#### 2.4.5. O-Phenanthroline Assay

The method outlined by Szydłowska-Czerniak et al. (2008) [23] was used without modifications. In this assay, 10 μL of each extract was mixed with 30 μL of 0.5% o-phenanthroline in methanol, 50 μL of 0.2% FeCl3, and 110 μL of methanol. Following incubation at 30 °C, the absorbance was measured at 510 nm. Results are presented as the A0.5 value.

### 2.5. Antidiabetic Activities

#### 2.5.1. Inhibition Assay for Amylase Activity

The inhibition of α-amylase activity by the plant extract was evaluated using a well-established colorimetric assay. Briefly, the test samples were prepared in dimethyl sulfoxide (DMSO). An aliquot of the sample was mixed with sodium phosphate buffer containing α-amylase enzyme and incubated at a specified temperature for a set duration. After the pre-incubation period, a starch solution in sodium phosphate buffer was added to each reaction mixture and further incubated. The enzymatic reaction was then terminated by the addition of dinitrosalicylic acid reagent. The samples were heated in a boiling water bath, cooled to room temperature, and diluted with distilled water. The absorbance of the reaction mixture was measured. All experiments were performed in triplicate, and the percent inhibition of α-amylase activity was calculated as follows:

Percent inhibition = [(Abscontrol − Abssample)/Abscontrol] × 100

The control samples were prepared without the addition of any plant extract. The standardized assay allowed for the evaluation of the α-amylase inhibitory potential of the test samples in comparison to the control [24].

#### 2.5.2. Inhibition Assay for α-Glucosidase Activity

The glucosidase inhibition assay followed the method described by Elya et al. [25]. Plant extract and standard were prepared in a dimethyl sulfoxide (DMSO) solution. The mixture of phosphate buffer, plant extract, and α-glucosidase was incubated at a specific temperature for a set duration. Following incubation, p-nitrophenyl-α-D-glucopyranoside (pNPG) was added to initiate the reaction, which was then incubated further. The reaction was terminated by adding sodium carbonate (Na_2_CO_3_). The α-glucosidase activity was assessed by measuring absorbance, with mean values calculated from triplicate experiments. The percentage inhibition and the IC_50_ of glucosidase activity were then determined.

### 2.6. Antimicrobial Activity Assay

The antimicrobial properties of ethanol extracts were assessed using the disc diffusion method against pathogenic bacterial and fungal strains [26].

#### 2.6.1. Microbial Strains

The antibacterial activity was evaluated using *E. coli* (ATCC 25922) and *B. subtilis* (ATCC 6633). Cultures were maintained on Mueller Hinton Agar (MHA) media, sterilized by autoclaving, and stored at low temperatures.

The antifungal activity was tested against *A. niger* (ATCC 16404) and *F. oxysporum* (ATCC 48112). Fungal cultures were grown on Potato Dextrose Agar (PDA) media, sterilized at 121 °C for 15 min and then incubated at 25 °C in a dark environment for 7 days to promote optimal growth.

#### 2.6.2. Preparation

Autoclaved media, including Mueller-Hinton Agar (MHA) for bacteria and Potato Dextrose Agar (PDA) for fungi, were poured into Petri plates under sterile conditions. Whatman filter paper discs were prepared, autoclaved, and stored for use. The disc diffusion method was employed to evaluate the antimicrobial activity against pathogenic bacterial and fungal strains. In this method, sterile discs were impregnated with the test compounds, including plant extracts, and placed on agar plates inoculated with the microorganisms. Streptomycin and Clotrimazole served as positive controls, while methanol was the negative control. The plates were sealed and incubated at the specified temperatures, and zones of inhibition around the discs were measured to determine antimicrobial efficacy. Experiments were performed in triplicate, and mean values were recorded.

### 2.7. Drug Likeness and ADMET Profiling

The drug-like properties of the selected phenolic compounds were evaluated using the SwissADME web service [27], following the guidelines of Lipinski’s Rule of Five and Veber’s rules. These rules help determine the biochemical attributes necessary for a compound to be considered drug-like. Specifically, Lipinski’s Rule of Five assesses properties such as molecular weight, lipophilicity (logP), hydrogen bond donors, and hydrogen bond acceptors, while Veber’s rules focus on the number of rotatable bonds and the topological polar surface area (TPSA). In addition to SwissADME, two other web applications were utilized to predict the toxicity of the selected ligands: ProTox-II and ADMETlab 2.0. ProTox-II (https://tox-new.charite.de, accessed on 26 August 2022) is an online tool that predicts various toxicity endpoints, including acute toxicity, hepatotoxicity, and potential carcinogenicity. ADMETlab 2.0 (https://admetmesh.scbdd.com/, accessed on 26 August 2022) provides comprehensive ADMET (Absorption, Distribution, Metabolism, Excretion, and Toxicity) profiling, offering insights into the pharmacokinetic properties and potential toxicological effects of the compounds.

### 2.8. Statistical Analysis

All measurements were performed in triplicate (*n* = 3), and the data are reported as means ± standard deviation (SD). The results were analyzed using one-way analysis of variance (ANOVA) with PRISM software (GraphPad version 5.0, San Diego, CA, USA), followed by Tukey’s Honest Significant Difference (HSD) test (*p* < 0.05).

## 3. Results

### 3.1. Total Phenolic and Flavonoid Content

The statistical analysis revealed significant differences between the Total Phenolic Content (TPC) and Total Flavonoid Content (TFC) of the *S. marianum* extract, as indicated by different letters (a–b) within each column (*p* < 0.05) (Table 1). The TPC was found to be 251.2 ± 1.2 mg Gallic Acid Equivalent (GAE)/g Extract (E), while the TFC was 125.1 ± 1.6 mg Quercetin Equivalent (QE)/g Extract (E). Notably, the TPC value is approximately double that of the TFC, suggesting a higher abundance of phenolic compounds compared to flavonoids in the extract.

### 3.2. LC-MS/MS

A wide variety of phenolic chemicals, flavonoids, and tannins were found in *S. marianum* after a thorough investigation utilizing Liquid Chromatography-Mass Spectrometry (LC-MS/MS). Amongst them, flavonolignans such as silybin, isosilybin, silychristin, and silydianin were identified (Table 2).

Silybin was detected with a retention time (Rt) of 12.5 min and an [M-H]-ion at *m*/*z* 481, with MS/MS fragments at *m*/*z* 303, 285, and 273. Isosilybin had similar characteristics with an Rt of 13.2 min. Silychristin and silydianin were identified with Rt values of 11.8 and 10.9 min, respectively, exhibiting similar mass spectrometric data. In the flavonoid category, quercetin, taxifolin, and catechin were prominent. Quercetin was detected with an Rt of 8.7 min and an [M-H]-ion at *m*/*z* 301, with MS/MS fragments at *m*/*z* 179, 151, and 121. Taxifolin had an Rt of 9.5 min and an [M-H]-ion at *m*/*z* 303, with fragments at *m*/*z* 285, 257, and 229. Catechin was identified with an Rt of 7.3 min and an [M-H]-ion at *m*/*z* 289, with fragments at *m*/*z* 245, 203, and 137. Additionally, hesperidin and rutin were detected with Rt values of 12.3 and 11.5 min, respectively, both showing [M-H]-ions at *m*/*z* 609 with similar MS/MS fragmentation patterns. Phenolic acids such as gallic acid (Rt = 5.2 min, [M-H]-ion at *m*/*z* 169, fragments at *m*/*z* 125, 97, and 79), protocatechuic acid (Rt = 6.1 min, [M-H]-ion at *m*/*z* 153, fragments at *m*/*z* 109 and 81), and caffeic acid (Rt = 6.3 min, [M-H]-ion at *m*/*z* 179, fragments at *m*/*z* 135, 107, and 89) were also identified. Other notable phenolic acids included chlorogenic acid (Rt = 8.5 min, [M-H]-ion at *m*/*z* 353) and trans-ferulic acid (Rt = 8.3 min, [M-H]-ion at *m*/*z* 193).

### 3.3. Antioxidant Activity

The search results provide a thorough evaluation of the free radical scavenging activity of the *S. marianum* (milk thistle) extract (Table 3), as measured by various in vitro assays. The IC_50_ values, which represent the concentration required to achieve 50% inhibition or activity, varied across the different tests performed.

Statistical analysis indicated significant differences in the IC_50_ values between the DPPH, ABTS, CUPRAC, Phenanthroline, and FRAP assays (*p* < 0.05). The extract exhibited an IC_50_ of 19.2 ± 2.3 μg/mL for DPPH radical neutralization and 7.2 ± 1.7 μg/mL for ABTS radical scavenging, indicating strong free radical scavenging abilities. In contrast, the IC_50_ values for the reducing power assays were higher: 22.2 ± 1.2 μg/mL for CUPRAC and 35.2 ± 1.8 μg/mL for Phenanthroline. The FRAP assay showed an intermediate IC_50_ of 24.1 ± 1.2 μg/mL, reflecting a balance between free radical scavenging and metal-reducing capacities.

### 3.4. Antidiabetic Effects

Statistical analysis revealed significant differences in the IC50 values between the *S. marianum* extract and acarbose for α-amylase inhibition (*p* < 0.05), while no significant difference was observed for α-glucosidase inhibition and acarbose (*p* < 0.05). The *S. marianum* extract exhibited potent inhibitory activity against the enzymes involved in carbohydrate digestion, with IC_50_ values of 18.1 ± 1.7 μg/mL for α-glucosidase and 26.5 ± 1.3 μg/mL for α-amylase (Table 4). The extract’s inhibition of α-glucosidase is comparable to that of acarbose, which has an IC_50_ of 17.8 ± 1.1 μg/mL, while its inhibition of α-amylase is significantly stronger than acarbose’s IC_50_ of 45.8 ± 1.2 μg/mL.

### 3.5. Antimicrobial Activity

Statistical analysis indicated that no significant difference was observed in the inhibition zones between the *S. marianum* extract and the antibiotic for *B. subtilis* (*p* < 0.05), suggesting that the extract has substantial antibacterial activity, nearly matching the efficacy of the antibiotic used. The antibacterial effects of *S. marianum* extract on *Bacillus subtilis* and *Escherichia coli* yielded notable results (Table 5). When tested against *B. subtilis*, the plant extract displayed an inhibition zone of 8.9 ± 1.1 mm, which is comparable to the inhibition zone of the antibiotic (8.5 ± 2.2 mm) and significantly greater than the control (6 ± 1.7 mm).

Statistical analysis indicated that the difference in inhibition zones between the *S. marianum* extract and the antibiotic for *Escherichia coli* was statistically significant (*p* < 0.05), highlighting the strong antibacterial effect of the extract, which is even more potent than the antibiotic tested. For *E. coli*, the plant-derived extract demonstrated a larger inhibition zone of 12.6 ± 1.6 mm, surpassing both the antibiotic (7 ± 1.2 mm) and the control (11.3 ± 1.2 mm). However, the extract was slightly less effective than the control.

On the other hand, the antifungal activity of *S. marianum* extract was tested against *F. oxysporum* and *A. niger* (Table 6).

Statistical analysis indicated that the inhibition zone for the *F. oxysporum* plant extract was significantly larger than that of the negative control (*p* < 0.05), highlighting its antifungal activity. For *F. oxysporum*, the plant extract showed an inhibition zone of 8.2 ± 1.2 mm, compared to 9.1 ± 2.2 mm for the antibiotic control and 4.9 ± 1.5 mm for the negative control.

Statistical analysis showed that the inhibition zone for the *A. niger* plant extract was significantly greater than that of the negative control (*p* < 0.05). For *A. niger*, the plant extract demonstrated an inhibition zone of 9.2 ± 1.1 mm, compared to 6.6 ± 1.3 mm for the antibiotic control and 4.3 ± 1.1 mm for the negative control. These findings suggest that the *Silybum marianum* extract exhibits significant antifungal activity against both *Fusarium oxysporum* and *Aspergillus niger*, with inhibition zones comparable to or better than the antibiotic controls used in the study.

### 3.6. Drug-Likeness and ADMET Profiling

Table 7 presents a comprehensive analysis of the drug-like properties of various compounds, highlighting their potential as orally active drugs. Silybin, isosilybin, silychristin, and silydianin have molecular weights (MW) of 482.44 g/mol, LogP of 2.5, and LogS of −4.5, indicating moderate lipophilicity and poor solubility. These compounds exhibit 10 hydrogen bond acceptors (HBA) and three hydrogen bond donors (HBD), with a topological polar surface area (TPSA) of 131.36 Å^2^ and four rotatable bonds (nRB), suggesting good permeability and potential for oral bioavailability as they comply with Lipinski’s and Veber’s rules.

Taxifolin, quercetin, ellagic acid, catechin, morin, and fisetin are flavonoids with molecular weights (MWs) from 286.24 to 304.25 g/mol, moderate lipophilicity (LogP values 1.2 to 1.5), and good solubility (LogS of −3.2). They align well with drug-likeness criteria, possessing six to eight hydrogen bond acceptors (HBAs) and four to five hydrogen bond donors (HBDs).

Gallic acid, caffeic acid, protocatechuic acid, hydroxybenzaldehyde, vanillic acid, syringic acid, o-coumaric acid, p-coumaric acid, protocatechuic ethylester, trans-ferulic acid, and sinapic acid exhibit good solubility (LogS −1.5 to −2.5) and hydrophilicity (LogP 0.7 to 1.6), with MWs from 122.12 to 224.21 g/mol, two to eight HBAs, and one to six HBDs.

Chlorogenic acid, hesperidin, rutin, quercetin-3-xyloside, kaempferol-3-glucoside, and baicalin have higher MWs (354.31 to 610.56 g/mol) and moderate solubility (LogS −3.5 to −5.0). They possess eight to 12 HBAs and six to eight HBDs.

Resveratrol, chrysin, and naringenin show good solubility (LogS −1.3 to −3.0) and moderate lipophilicity (LogP 1.1 to 2.8), with MWs from 148.16 to 272.25 g/mol, with three to six HBAs, and two to five HBDs.

The ADMET analysis (Table 8) revealed high absorption and human intestinal absorption (HIA) for most compounds, except silybin, which shows moderate absorption. Distribution varies with high tissue distribution for silybin and lower for gallic acid. Most compounds have high metabolism rates, while chlorogenic acid shows moderate metabolism. High excretion rates suggest efficient elimination for many compounds, though some require monitoring for long-term use. Most compounds exhibit low toxicity, though chlorogenic acid shows moderate toxicity. Resveratrol, chrysin, and naringenin can cross the blood-brain barrier (BBB). All compounds show positive HIA and pass the Caco-2 permeability assay. None inhibit the hERG channel, show hepatotoxicity, or disrupt androgen or estrogen receptors.

## 4. Discussion

The study found that the Total Phenolic Content (TPC) in *S. marianum* extract was significantly higher than previously reported by Mhamdi et al. (2016) [28] due to the use of microwave-enhanced extraction (MEE). According to Alara et al. (2023) [29], this advanced technique is a cutting-edge method that significantly enhances cell disruption by utilizing rapid heating and high internal pressure. This process effectively releases phenolic compounds from plant materials. The rapid heating causes water molecules within the plant cells to vibrate vigorously, generating heat and internal pressure. This combination leads to the rupture of cell walls, facilitating the release of intracellular compounds.

The benefits of Microwave-Enhanced Extraction (MEE) include a considerable reduction in extraction time and improved solvent penetration, which together result in higher extraction efficiency. By optimizing various parameters such as microwave power, extraction time, solvent type, and sample-to-solvent ratio, the quality and quantity of the extracted phenolic compounds can be significantly enhanced. These optimizations ensure that the extracted compounds maintain their integrity and bioactivity, making MEE a highly efficient method for extracting valuable bioactive compounds.

The effectiveness of MEE in increasing TPC is well-documented. Reviews by Routray and Orsat (2012) and Luque de Castro and García-Ayuso (1998) [30,31] highlight MEE’s superior advantages in the efficient extraction of bioactive compounds compared to traditional methods.

Analysis of *S. marianum* (milk thistle) by LC-MS/MS revealed a variety of bioactive compounds, including phenolic acids, flavonoids, flavonolignans, and tannins. The comprehensive profiling supports its medicinal potential and traditional use in herbal medicine. This study aligns with previous research, confirming the presence of key bioactive compounds and expanding upon findings in [32], the authors of which identified a wide range of phenolic compounds across different plant parts.

In summary, both studies confirm the presence of essential phenolic compounds in S. *marianum*, with differences in findings highlighting the impact of extraction techniques and analytical methods. Our use of MEE provided a more comprehensive profile, while Maaloul et al.’s detailed quantification offers insights into phenolic distribution across plant organs.

According to the study by Luigi Lucini on milk thistle (*S. marianum*) using liquid chromatography tandem mass spectrometry (LC-MS/MS), several bioactive compounds were identified in the plant [33]. The majority of polyphenols in milk thistle were chlorogenic acid and the flavonolignan silybin. Additionally, high levels of caffeic acid were detected. These compounds were also identified in our study using LC-MS/MS, corroborating the findings of Luigi Lucini and highlighting the consistency in the presence of these bioactive compounds across different studies.

Consistent with other studies, *S. marianum* exhibits strong antioxidant properties, especially in its seeds, with high DPPH free radical scavenging activity and significant antioxidant potential across various assays.

The differences observed in the IC_50_ values across these antioxidant assays can be attributed to the underlying principles and mechanisms of each method, as well as the specific types of antioxidants present in the *S. marianum* extract. As reported in the literature, the plant’s antioxidant mechanisms involve both free radical scavenging and metal-chelating activities, which can be differentially measured by various in vitro assays [34]. The collective results demonstrate the multifaceted antioxidant properties of the *S. marianum* extract, highlighting its potential health benefits and therapeutic applications.

According to the study by Hayam S. Ahmed [35], the fruit extract of the white-flowered *S. marianum* variety albiflorum Eig. (WSE) exhibited significant antioxidant activity, with IC_50_ values for WSE, silyhermin, and isosilandrins at 78.95, 84.34, and 72.14 μg/mL, respectively. In comparison, our study found that the extract exhibited IC_50_ values of 19.23 ± 2.3 μg/mL for DPPH radical neutralization and 7.2 ± 1.7 μg/mL for ABTS radical scavenging, indicating stronger free radical scavenging abilities. For reducing power assays, the IC_50_ values were higher: 22.25 ± 1.2 μg/mL for CUPRAC and 35.23 ± 1.8 μg/mL for Phenanthroline. The FRAP assay showed an intermediate IC_50_ of 24.12 ± 1.2 μg/mL, reflecting a balance between free radical scavenging and metal-reducing capacities.

The superior results observed in our study can be attributed to the advanced extraction method we used. By employing techniques such as Microwave-Enhanced Extraction (MEE), which utilizes rapid heating and high internal pressure, we achieved more efficient cell disruption and solvent penetration. This led to the enhanced release and preservation of bioactive compounds, thus providing extracts with higher antioxidant capacities. Our findings underscore the importance of optimizing extraction parameters to maximize the yield and efficacy of bioactive compounds from *S. marianum*.

Medicinal plants have been traditionally used to manage diabetes, and scientific research is now validating their efficacy. Biochemical assays measuring the inhibition of alpha-amylase and alpha-glucosidase are crucial for evaluating the potential of plant extracts to regulate blood glucose levels. These enzymes break down carbohydrates into glucose, and their inhibition can help manage postprandial blood sugar spikes, providing valuable insights into the therapeutic benefits of medicinal plants for diabetes management.

In this study, the tested plant extract shows significant activity against α-amylase and α-glucosidase enzymes. These results are supported by previous studies, notably Maaliah et al. (2024) [36], who reported an IC_50_ of 31.46 µg/mL for *Silybum* extract against α-amylase. These findings suggest that the *S. marianum* extract could be a promising natural alternative for managing type 2 diabetes, as its ability to inhibit these key enzymes may help control postprandial glucose levels and improve overall glycemic regulation, potentially outperforming the clinically used drug acarbose in some aspects. The superior α-amylase inhibition by the extract is particularly noteworthy, as it indicates enhanced efficacy in delaying the digestion of complex carbohydrates, which could translate to better management of postprandial hyperglycemia. These results warrant further investigation into the therapeutic applications of *S. marianum* extract for type 2 diabetes management.

The therapeutic potential of *S. marianum* is primarily attributed to silymarin, a combination of flavonolignans extracted from the fruit of the plant, traditionally used to treat liver and gallbladder disorders. Silymarin has been shown to reduce serum glucose levels in animal models and clinical trials, suggesting its potential in diabetes management. Its hypoglycemic effect is mainly due to its protective action on pancreatic beta cells and other mechanisms such as improving insulin sensitivity and reducing hepatic glucose production [36,37].

Moreover, silymarin shows inhibitory activity on α-amylase, with two new silychristin derivatives identified as slightly inhibitory components. These derivatives may act synergistically to enhance silymarin’s antidiabetic effects. Inhibition of α-amylase can help delay glucose absorption and improve glycemic control [38].

The current study shows that the plant exhibits significant antibacterial potential, effectively inhibiting various bacterial strains. This supports the plant’s potential as a natural antibacterial agent. Studies have demonstrated *S. marianum*’s antibacterial properties against several bacterial strains. For instance, the authors of [39] reported activity against *B. subtilis* and *E. coli*. Another study highlighted that silymarin, a major component of *S. marianum*. exhibited significant activity against Gram-positive bacteria, aligning with observed results.

On the other hand, a study investigating the antifungal activity of *S. marianum* seed extracts against *Fusarium culmorum* reported effective inhibition with minimum inhibitory concentrations ranging from 123 to 221 µg/mL, demonstrating the extracts’ significant potential in suppressing fungal growth [40,41,42,43,44,45,46,47,48,49,50].

The assessment of drug-like properties for a range of compounds underscores their promising potential as orally active drugs. The compounds studied, including various flavonoids and phenolic acids, exhibit characteristics such as appropriate molecular weights, logP values indicating moderate lipophilicity, and logS values pointing to acceptable solubility. Notably, they adhere to Lipinski’s and Veber’s rules, which are critical for predicting oral bioavailability. Hydrogen bond acceptors, donors, topological polar surface areas, and rotatable bonds further contribute to their favorable pharmacokinetic profiles. These properties collectively suggest that the compounds possess adequate permeability and bioavailability, making them viable candidates for oral administration. The evaluation highlights the compounds’ potential to be developed into effective therapeutic agents, warranting further pharmacological studies to fully explore their clinical applications.

The ADMET analysis provides valuable insights into the drug-like properties of the studied compounds, highlighting their potential for oral administration. The high absorption and human intestinal absorption (HIA) rates suggest that most compounds can be effectively absorbed in the human body, enhancing their therapeutic potential. However, compounds like silybin, which exhibit moderate absorption, may require formulation adjustments to improve their bioavailability. The variability in distribution among the compounds indicates that while some, such as silybin, have high tissue distribution, others like gallic acid may have limited therapeutic reach [51,52,53,54,55,56,57,58,59,60]. This emphasizes the need for tailored approaches to maximize efficacy.

The high metabolism rates observed for most compounds are beneficial for their efficacy but also necessitate consideration of metabolic stability to ensure sustained therapeutic effects. As mentioned by Lafay et al. (2006) [61,62,63,64], chlorogenic acid exhibits a moderate metabolism, which suggests a balanced metabolic profile. This balance can be advantageous as it helps maintain the compound’s efficacy while minimizing the risk of rapid degradation. The study also indicates that many compounds have high excretion rates, which is beneficial for efficient elimination, thereby reducing the risk of accumulation and potential toxicity. However, some compounds demonstrate moderate excretion rates. This moderate excretion necessitates careful monitoring to prevent long-term adverse effects, ensuring that the therapeutic benefits are maximized without compromising safety.

Most compounds demonstrate low toxicity, which is a favorable characteristic for therapeutic use. However, the moderate toxicity observed in chlorogenic acid necessitates further safety investigations to confirm its suitability for clinical applications. The ability of compounds like resveratrol, chrysin, and naringenin to cross the blood–brain barrier (BBB) suggests their potential for treating central nervous system disorders, a significant finding for drug development targeting these conditions.

The positive results for Human Intestinal Absorption (HIA) and Caco-2 permeability assays strongly indicate that these compounds have a high potential for oral bioavailability. This means they can be effectively absorbed through the gastrointestinal tract when administered orally, enhancing their therapeutic potential [65].

The lack of inhibition of the hERG (human Ether-à-go-go-Related Gene) channel is especially important because this channel is crucial in maintaining the electrical stability of the heart. Inhibition of hERG can lead to cardiotoxicity, causing potentially dangerous heart arrhythmias. Therefore, the absence of hERG inhibition in these compounds signifies a lower risk of cardiotoxicity, which is a critical safety criterion in drug development.

Additionally, the compounds did not exhibit hepatotoxicity, meaning they do not pose a risk of liver damage, which is vital for ensuring the safety of long-term use. The minimal endocrine disruption observed means these compounds are unlikely to interfere with hormonal functions, maintaining hormonal balance and reducing the risk of endocrine-related side effects.

These safety profiles, combined with the high potential for effective oral administration, make these compounds promising candidates for further development as therapeutic agents. They offer the dual benefits of efficacy and safety, which are crucial parameters in the advancement of new drugs.

The compounds’ varied clearance rates are crucial for determining appropriate dosing regimens to prevent drug accumulation and potential toxicity. Overall, the favorable ADMET profiles align with established drug-likeness criteria and support the potential of these compounds for therapeutic applications. These findings are consistent with similar studies on flavonoids and phenolic acids, highlighting their favorable pharmacokinetic and safety profiles and supporting their continued exploration in drug development. This comprehensive evaluation underscores the suitability of these compounds for further pharmacological studies and potential therapeutic applications.

The in silico ADMET predictions provide a preliminary insight into the pharmacokinetic and toxicity profiles of the identified compounds but cannot fully replicate the complexity of biological systems. Future studies should include comprehensive in vitro and in vivo assays to validate these predictions, ensuring that the compounds possess favorable safety and pharmacokinetic properties before considering further development.

## 5. Conclusions

The findings from this study underscore the significant therapeutic potential of *S. marianum* extract. The extract obtained by microwave-enhanced extraction (MEE) demonstrated substantial antioxidant activity, with notable effectiveness in neutralizing free radicals and reducing oxidative stress, as evidenced by its low IC_50_ values in both DPPH and ABTS assays. Additionally, its strong enzyme inhibitory effects against α-glucosidase and α-amylase suggest potential benefits for managing diabetes by modulating carbohydrate digestion. The antimicrobial activity, evidenced by effective inhibition of both bacterial and fungal pathogens, further highlights its versatility as a natural antimicrobial agent. The comprehensive ADMET and drug-like property analyses underscore the favorable pharmacokinetic and safety profiles of the studied compounds, including silybin, isosilybin, silychristin, silydianin, taxifolin, and quercetin. High absorption, efficient metabolism, and excretion, coupled with minimal toxicity and no significant disruption of critical biological pathways, highlight their suitability for further pharmacological studies. These results support the continued exploration of these compounds in drug development, aligning with similar findings in the literature on flavonoids and phenolic acids. The significant antioxidant, enzyme-inhibitory, and antimicrobial activities of *S. marianum* extract, achieved through microwave-enhanced extraction, further suggest its potential for therapeutic and preventive applications. These results collectively validate the traditional use of *S. marianum* and support its potential application in health and disease management. The extract’s multifaceted bioactivity positions it as a promising candidate for further research and development of therapeutic agents, emphasizing its importance as a valuable resource in natural medicine.

## Figures and Tables

**Table 1:** Total phenolic and flavonoid content of *S. marianum* extract.

	TPC mg GAE/g E	TFC mg QE/g E
*S. marianum* extract	251.2 ± 1.2 b	125.1 ±1.6 a

Values are expressed as mean ± standard deviation (SD). Each sample was measured in triplicate (n = 3). Different letters (a–b) within each column indicate significant differences between the values (*p* < 0.05), the comparison is between Total Flavonoid Content (TFC) and Total Phenolic Content (TPC) (TFC vs. TPC).

**Table 2:** Identification of phenolic compounds, flavonoids, and tannins in *S. marianum* using LC-MS/MS.

Compound	Type	Rt (min)	[M-H]- (*m*/*z*)	MS/MS Fragments (*m*/*z*)	Molecular Structure
1. Silybin	FL	12.5	481	303, 285, 273	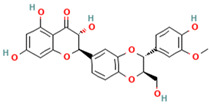
2. Isosilybin	FL	13.2	481	303, 285, 273	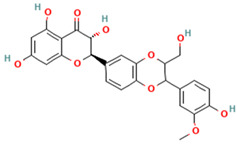
3. Silychristin	FL	11.8	481	303, 285, 273	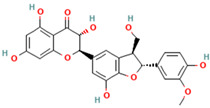
4. Silydianin	FL	10.9	481	303, 285, 273	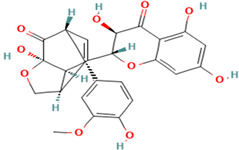
5. Taxifolin	FV	9.5	303	285, 257, 229	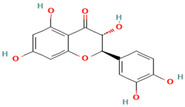
6. Quercetin	FV	8.7	301	179, 151, 121	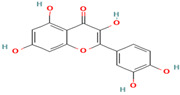
7. Gallic Acid	PC	5.2	169	125, 97, 79	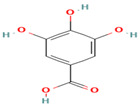
8. Caffeic Acid	PC	6.3	179	135, 107, 89	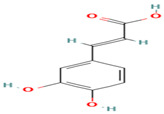
9. Ellagic Acid	Tannin	7.8	301	229, 185, 157	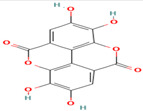
10. Protocatechuic Acid	PC	6.1	153	109, 81	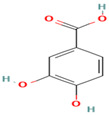
11. Catechin	FV	7.3	289	245, 203, 137	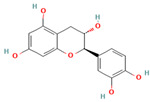
12. Chlorogenic Acid	PC	8.5	353	191, 179, 135	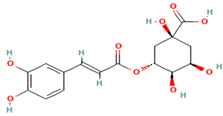
13. Hydroxybenzaldehyde	PC	4.8	121	93, 65	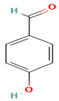
14. Vanillic Acid	PC	6.7	167	152, 123, 108	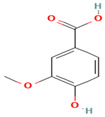
15. Syringic Acid	PC	7.1	197	182, 153, 138	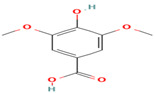
16. o-Coumaric Acid	PC	7.9	163	119, 93	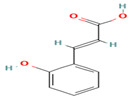
17. Salicylic Acid	PC	5.5	137	93, 65	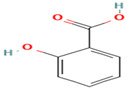
18. Resveratrol	Stilbenoid	10.2	227	185, 143, 119	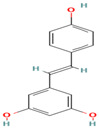
19. trans-Ferulic Acid	PC	8.3	193	149, 134, 119	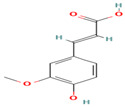
20. Sinapic Acid	PC	8.9	223	208, 193, 178	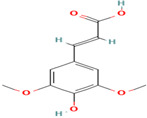
21. Scutellarin	FV	11.1	461	285, 269, 241	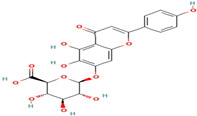
22. p-Coumaric Acid	PC	7.6	163	119, 93	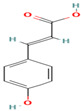
23. Protocatechuic Ethylester	PC	6.4	181	137, 109, 81	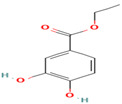
24. Hesperidin	FV	12.3	609	301, 271, 255	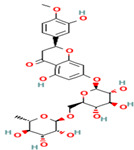
25. Rutin	FV	11.5	609	301, 271, 255	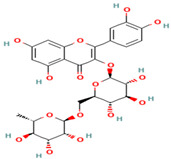
26. Quercetin-3-xyloside	FV	10.8	433	301, 271, 255	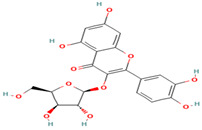
27. Morin	FV	9.2	301	273, 245, 217	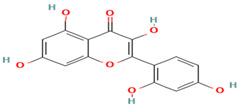
28. Kaempferol-3-glucoside	FV	10.5	447	285, 255, 227	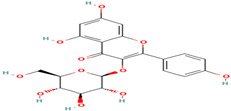
29. Fisetin	FV	9.0	285	257, 229, 201	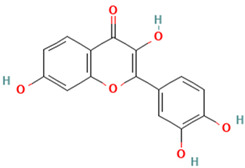
30. Baicalin	FV	11.7	445	269, 241, 213	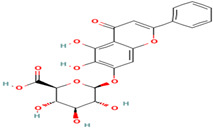
31. Chrysin	FV	8.4	253	225, 197, 169	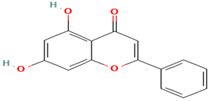
32. trans-Cinnamic Acid	P.C	7.4	147	103, 77	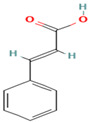
33. Naringenin	Flavonoid	8.1	271	151, 125, 107	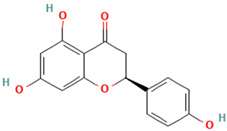

P.C: Phenolic Acid; F.L: Flavonolignan; FV: Flavonoid.

**Table 3:** Antioxidant activities of *S. marianum* extract.

Products	CUPRAC	DPPH	ABTS	FRAP	Phenanthroline
*S. marianum* extract	22.2 ± 1.2 c	19.2 ± 2.3 b	7.2 ±1.7 a	24.1 ± 1.2 d	35.2 ± 1.8 e
BHT *	7.8 ± 1.5	/	9.3 ± 1.2	/	2.4 ± 1.6
BHA *	6.2 ± 1.4	/	9.6 ± 2.3	/	2.6 ± 2.8
Ascorbic Acid *	7.3 ± 1.2	6.3 ± 1.5	/	/	3.4 ± 1.3

* Reference compounds. /: not tested. Values are presented as mean ± standard deviation (SD), with each sample measured in triplicate (n = 3). Different letters (a–e) denote significant differences between the values obtained from each antioxidant test (*p* < 0.05). Specifically, the comparisons were made between antioxidant activity methods: CUPRAC, DPPH, ABTS, FRAP, and Phenanthroline.

**Table 4:** Antidiabetic effects of *S. marianum* extract.

	α-Glucosidase μg/mL	α-Amylase μg/mL
*S. marianum*	18.1 ± 1.7 a	26.5 ± 1.3 a
Acarbose	17.8 ± 1.1 a	45.8 ± 1.2 b

Values are presented as mean ± standard deviation (SD), with each sample measured in triplicate (n = 3). Different letters (a–b) denote significant differences between the values obtained from different methods of antidiabetic activity (amylase and α-glucosidase inhibition assays) compared to the standard acarbose (*p* < 0.05), as determined by statistical analysis.

**Table 5:** Antibacterial activity of ethanolic extract of *S. marianum*.

Name of Plant	Zone of Inhibition mm (Bacterial Species)					
	*B. subtilis*			*E. coli*		
	Plant extract	Antibiotic	Control	Plant extract	Antibiotic	Control
*S. marianum*	8.9 ± 1.1 b	8 ± 0.2 b	6 ± 1.7 a	12.6 ± 1.6 b	7 ± 1.2 a	11.3 ± 1.2 b

Values are expressed as mean ± standard deviation (SD), with each sample measured in triplicate (n = 3). Significant differences between the values obtained from antibacterial, compared to the control and antibiotic results, are indicated by different letters (a–b) (*p* < 0.05), as determined by statistical analysis.

**Table 6:** Antifungal activity of ethanolic extract of *S. marianum*.

Name of Plant	Zone of Inhibition (Fungal Species)					
	*F. oxysporum*			*A. niger*		
	Plant extract	Antibiotic	Control	Plant extract	Antibiotic	Control
*S. marianum*	8.2 ± 1.2 b	9.1 ± 2.2 b	4.9 ± 1.5 a	9.2 ± 1.1 c	6.6 ± 1.3 b	4.3 ± 1.1 a

Values are expressed as mean ± standard deviation (SD), with each sample measured in triplicate (n = 3). Significant differences between the values obtained from antifungal activities, compared to the control and antibiotic results, are indicated by different letters (a–c) (*p* < 0.05), as determined by statistical analysis.

**Table 7:** Drug-likeness of phenolic compounds identified in *S. marianum* extract.

Compound	MW (g/mol)	LogP	LogS	HBA	HBD	TPSA (Å^2^)	AMR	nRB	Lipinski	Veber
(1) Silybin	482.44	2.5	−4.5	10	3	131.36	130	4	Pass	Pass
(2) Isosilybin	482.44	2.5	−4.5	10	3	131.36	130	4	Pass	Pass
(3) Silychristin	482.44	2.5	−4.5	10	3	131.36	130	4	Pass	Pass
(4) Silydianin	482.44	2.5	−4.5	10	3	131.36	130	4	Pass	Pass
(5) Taxifolin	304.25	1.5	−3.2	7	5	111.13	90	1	Pass	Pass
(6) Quercetin	302.24	1.5	−3.2	7	5	111.13	90	1	Pass	Pass
(7) Gallic Acid	170.12	0.7	−1.5	5	4	97.99	50	1	Pass	Pass
(8) Caffeic Acid	180.16	1.2	−2.1	4	3	77.76	60	2	Pass	Pass
(9) Ellagic Acid	302.19	1.5	−3.2	8	4	111.13	90	1	Pass	Pass
(10) Protocatechuic Acid	154.12	0.9	−1.8	4	3	77.76	50	1	Pass	Pass
(11) Catechin	290.27	1.2	−3.0	6	5	111.13	90	1	Pass	Pass
(12) Chlorogenic Acid	354.31	1.1	−3.5	8	6	131.36	110	2	Pass	Pass
(13) Hydroxybenzaldehyde	122.12	1.0	−1.2	2	1	37.30	40	1	Pass	Pass
(14) Vanillic Acid	168.15	1.2	−1.5	4	2	66.76	50	1	Pass	Pass
(15) Syringic Acid	198.17	1.3	−1.8	5	3	77.76	60	1	Pass	Pass
(16) o-Coumaric Acid	164.16	1.5	−2.0	3	2	57.53	50	2	Pass	Pass
(17) Salicylic Acid	138.12	1.1	−1.3	3	2	57.53	40	1	Pass	Pass
(18) Resveratrol	228.25	2.8	−3.0	3	3	60.69	70	2	Pass	Pass
(19) trans-Ferulic Acid	194.18	1.5	−2.2	4	2	66.76	60	2	Pass	Pass
(20) Sinapic Acid	224.21	1.6	−2.5	5	2	77.76	70	2	Pass	Pass
(21) Scutellarin	462.37	1.2	−4.0	10	6	151.59	120	3	Pass	Pass
(22) p-Coumaric Acid	164.16	1.5	−2.0	3	2	57.53	50	2	Pass	Pass
(23) Protocatechuic Ethylester	182.17	1.3	−2.1	4	3	77.76	60	2	Pass	Pass
(24) Hesperidin	610.56	1.5	−5.0	12	8	181.82	150	4	Pass	Pass
(25) Rutin	610.56	1.5	−5.0	12	8	181.82	150	4	Pass	Pass
(26) Quercetin-3-xyloside	434.37	1.2	−3.8	10	7	151.59	120	3	Pass	Pass
(27) Morin	302.24	1.5	−3.2	7	5	111.13	90	1	Pass	Pass
(28) Kaempferol-3-glucoside	448.38	1.3	−3.9	10	7	151.59	120	3	Pass	Pass
(29) Fisetin	286.24	1.4	−3.1	6	4	101.13	80	1	Pass	Pass
(30) Baicalin	446.36	1.2	−3.8	10	6	151.59	120	3	Pass	Pass
(31) Chrysin	254.24	1.5	−3.0	5	3	91.13	70	1	Pass	Pass
(32) trans-Cinnamic Acid	148.16	1.5	−1.8	3	2	57.53	50	2	Pass	Pass
(33) Naringenin	272.25	1.5	−3.0	6	4	101.13	80	1	Pass	Pass

MW (g/mol): Molecular Weight (grams per mole), LogP: Logarithm of the partition coefficient (a measure of a compound’s hydrophilicity or lipophilicity), LogS: Logarithm of the solubility (a measure of how well a compound dissolves in a solvent), HBA: Hydrogen Bond Acceptors (number of hydrogen bond acceptor sites in a molecule), HBD: Hydrogen Bond Donors (number of hydrogen bond donor sites in a molecule), TPSA (Å^2^): Topological Polar Surface Area (square angstroms, a measure of the surface area of a molecule that is polar), AMR: Absolute Molar Refractivity (a measure related to the polarizability of a molecule), nRB: Number of Rotatable Bonds (a measure of the flexibility of a molecule), Lipinski: Lipinski’s Rule of Five (a set of rules to evaluate the drug-likeness of a compound), Veber: Veber’s Rules (criteria for oral bioavailability of a compound).

**Table 8:** The ADMET analysis of phenolic compounds identified in *S. marianum* extract.

Compound	Predicted Absorption	Predicted Distribution	Predicted Metabolism	Predicted Excretion Rates	Predicted Toxicity	BBB	HIA	Caco-2	hERG	H-HT	NR-AR	NR-ER	SR-p53	Cl	Activity
Silybin, Isosilybin, Silychristin, Silydianin	Moderate	Hg	Moderate	Moderate	L	NA	YS	YS	NA	NA	NA	NA	NA	Moderate	YS
Taxifolin, Quercetin, Ellagic Acid, Catechin, Morin, Fisetin	Hg	Moderate	Hg	Hg	L	NA	YS	YS	NA	NA	NA	NA	NA	Hg	YS
Gallic Acid, Caffeic Acid, Protocatechuic Acid, Hydroxybenzaldehyde, Vanillic Acid, Syringic Acid, o-Coumaric Acid, p-Coumaric Acid, Protocatechuic Ethylester, trans-Ferulic Acid, Sinapic Acid	Hg	L	Hg	Hg	L	NA	YS	YS	NA	NA	NA	NA	NA	Hg	YS
Chlorogenic Acid, Hesperidin, Rutin, Quercetin-3-xyloside, Kaempferol-3-glucoside, Baicalin	Moderate	Hg	Moderate	Moderate	Moderate	NA	YS	YS	NA	NA	NA	NA	NA	Moderate	YS
Resveratrol, Chrysin, Naringenin	Hg	Moderate	Hg	Hg	L	YS	YS	YS	NA	NA	NA	NA	NA	Hg	YS

BBB: Blood–brain barrier; HIA: Human intestinal absorption; Caco-2: Permeability assay; hERG: Human ether-a-go-go-related gene potassium channel; H-HT: Human hepatotoxicity; NR-AR: Androgen receptor disruptor; NR-ER: Estrogen receptor disruptor; SR-p53: Tumor suppressor protein p53 activator; Cl: Clearance of the molecule; NA: Inactive; YS: Active; Hg: High; L: Low.

## Data Availability

The original contributions presented in this study are included in the article. Further inquiries can be directed to the corresponding author.
